# Multi-analyte approach for determining the extraction of tobacco constituents from pouched snus by consumers during use

**DOI:** 10.1186/1752-153X-7-55

**Published:** 2013-04-02

**Authors:** Helena Digard, Nathan Gale, Graham Errington, Nicola Peters, Kevin McAdam

**Affiliations:** 1 Group R&D, British American Tobacco (Investments) Ltd., Southampton, SO15 8TL, United Kingdom

**Keywords:** Snus, Dosimetry, Exposure assessment, Multi-analyte chemical analysis, Nicotine, Tobacco-specific nitrosamines

## Abstract

**Background:**

Snus is a smokeless oral tobacco product with a significant history of use in Sweden, where it is regulated under food legislation. Users place a small porous sachet or a pinch of loose snus between the upper jaw and cheek for approximately one hour, leading to partial intake of tobacco constituents. To understand user exposure to tobacco, a multi-analyte approach based on the extraction of pouches by methanol, ethanol and water was validated and applied to the measurement of various constituents, including nicotine, four tobacco-specific nitrosamines (TSNAs), propylene glycol, water, ammonium, nitrate, sodium, chloride, linalool, citronellol, linalyl acetate and geraniol, extracted from snus pouches during use by human consumers.

**Results:**

After validation against established single-analyte methods, the multi-analyte approach was used to determine constituent levels in snus pouches before and after one hour of use. Although the concentrations in the snus pouches varied from nanogram (e.g. TSNAs) to milligram (e.g. nicotine, sodium and propylene glycol) quantities (25.1 ng to 35.3 mg per 1 g pouch), the mean percentage extracted varied only from 19.2% for linalyl acetate to 37.8% for the TSNA 4-(methylnitrosamino)-1-(3-pyridyl)-1-butanone (NNK) among all constituents analyzed. The TSNAs, some of which are known carcinogens, showed the highest percentage extraction (range 34.6%–37.8%). Measurement variability was low for all analytes, ranging from 2.4% (total TSNAs, NAT) to 9.5% (geraniol). By contrast, inter-subject variability ranged from 6.7% (NAB) to 52.2% (linalyl acetate), and was greater than 20% for eight of the constituents analyzed. Intra-subject variability ranged from 3.4% (citronellol) to 29.7% (geraniol).

**Conclusions:**

Generally, less than a third of each constituent tested was extracted during one hour of snus use, independent of constituent concentration. The variable nature of in-use extraction was shown to be driven by inter-subject variability. The results provide insight into possible mechanisms controlling constituent extraction in the mouth during snus use, and provide reference data for the development of in-vitro laboratory systems for estimating extraction of tobacco constituents from snus.

## Background

Smokeless tobacco products (STPs) have been identified as Group 1 (known) human carcinogens by the International Agency for Research on Cancer (IARC) [1]. Globally, however, there are several STP categories which differ in manufacture, content, and method (e.g. location in mouth), duration and frequency of use. When considering the diversity of STPs, the UK Royal College of Physicians concluded that different categories of STPs pose varying levels of risk to their users in line with their toxicant content [2].

Many studies have investigated the chemical content of STPs. Among the approximately 5000 chemicals identified in tobacco [3], IARC listed 28 chemical agents or carcinogens in STPs [1]. Subsequently, the Tobacco Products Scientific Advisory Committee of the Center for Tobacco Products at the US Food and Drug Administration (FDA) prioritized a draft initial list of 44 harmful or potentially harmful compounds (H/PHC) in STPs [4] which was developed into an established list of 93 H/PHC in tobacco and smoke [5]. The FDA recently proposed an abbreviated list of nine of these 44 compounds in STPs, amongst an abbreviated list of 20 constituents in tobacco and smoke, that are required to be measured annually and the levels reported by manufacturers [6].

Owing to considerable differences in patterns of usage of the various categories of STPs, it is possible that toxicant content alone is insufficient to characterize fully the risk profiles of STPs. For example, users of dissolvable STPs (such as “sticks”, “strips” and solid tablets designed to be placed in the oral cavity and allowed to dissolve during normal use) are likely to consume the whole STP. By contrast, chewing tobacco is placed in the saliva-rich environment between the lower gum and cheek prior to mastication, and users occasionally expectorate salivary STP leachates. Users of Swedish snus place either a small porous sachet or a pinch of loose snus in the relatively saliva-poor area between the upper jaw and cheek for an hour on average, and undertake relatively little mechanical work [7]. At the end of STP use, users of chewing tobaccos and snus discard the remaining tobacco portions from their mouths, leading to partial intake of the tobacco constituents. Hence, it is likely that there are significant differences in exposure to toxicants between users of STPs who consume all and those who consume part of the tobacco mass. Moreover, differences in salivary content, amount of mechanical work on the STP, and levels of expectoration may also affect exposure among users of different types of STP. Understanding these differences in exposure can provide valuable insight into the risk profiles of different STP categories.

Historically, four approaches have been used to estimate toxicant exposure in STP users. Pharmacokinetic studies examine the plasma levels of toxicants, other constituents (such as nicotine [8]) and their metabolites among STP users in a clinical setting. Although providing a robust examination of toxicant uptake and overall plasma concentrations, this technique is limited by the controlled and potentially atypical usage documented in an invasive clinical setting, the limited range of analytical methods for relevant species in plasma, and the expense and timescale for conducting such studies.

Clinical studies involving measurement of biomarkers of exposure in urine of STP users (e.g. biomarkers of 4-(methylnitrosamino)-1-(3-pyridyl)-1-butanone (NNK) [9] and nicotine [10]) have provided insight into toxicant exposure and effects on STP users. Unlike pharmacokinetic studies, these studies can accommodate normal usage of STPs; however, they also face limitations such as cost, relatively small numbers of validated biomarkers for STP toxicants and compliance issues during ambulatory studies.

In-vitro systems have also been used to estimate extraction of STP constituents (e.g. toxic metals [11]). The applicability of in-vitro approaches is currently restricted by difficulties in reproducing real-use factors such as usage time, mechanical work on the STP in the mouth of users, expectoration and the chemical composition of saliva in users.

The fourth approach is based on measurements of toxicant extraction from STPs after normal usage. In such studies, subjects use snus in a normal or timed manner [10,12-14]. Chemical analysis of each portion after use, and comparison with the content of matched unused snus portions, allows estimation of the amount of constituents extracted during snus use. This last approach provides a flexible way of estimating toxicant exposure in STP users because any tobacco constituent for which an analytical method can be developed is within its scope. Normal usage patterns can be accommodated within the study design, and the non-invasive nature of the approach means that comparatively large numbers of users, products and constituents can be accommodated.

Although the above approaches have begun to establish the level of extraction of constituents from STPs, the range of constituents studied has been small and, more importantly, detailed analytical methods and validation data are lacking for extraction studies. As a result, the aim of this study was to examine the extraction of constituents from Swedish pouched snus by users during normal consumption as evaluated by a survey of snus usage among regular users. The robustness of the technique was validated for several constituents and then applied to a pilot study among users of Swedish pouched snus. A range of constituents, including known toxicants, common ions, and both water-soluble and sparingly water-soluble compounds, were analyzed with the aim of understanding factors influencing their extraction such as solubility. Species examined included those endogenous to tobacco, such as nicotine, and exogenous species such as the low solubility terpenoid aroma compounds linalyl acetate and linalool, typically found in Swedish-style snus. Product, analytical, and inter- and intra-subject variability was determined, enabling a discussion of effective study design for future studies of this kind.

## Experimental

### Survey of snus users to determine patterns of usage

Responses to a number of questions from a 2007/2008 telephone survey [7] were used to identify appropriate parameters for the subsequent extraction study.

### Collection of used snus samples to determine constituent extraction

#### Study population

Extraction studies were conducted in Sweden between August and October 2008 by an established consumer research agency, GfK Sverige AB (Sweden), a member of the Association of Swedish Market Research Institutes (SMIF). The studies were carried out in accordance with both the regulations established by SMIF and the International Chamber of Commerce/European Society for Opinion and Marketing Research (ICC/ESOMAR) Code on Market and Social Research. Via a telephone recruitment survey, 21 male users of pouched snus, aged between 19 and 64 years (mean 35 years, median 33 years) and living in the Stockholm or Lund area, were selected on the basis of normal use of a minimum of eight 1 g snus pouches per day for at least 1 hour per pouch. Each potential subject was asked to state any snus brand that they disliked; those who included the study brand in their response were not recruited. Subjects provided informed consent and were paid for their involvement.

#### Study product

The most commonly used snus pouch size and style in Sweden is 1 g “brown”-style (i.e. with water content in the region of 50%) [7]. Lucky Strike Original pouched snus (LS Brown) was chosen as an example of this format, with known chemical composition. The product was purchased from Fiedler & Lundgren (Malmo, Sweden), repacked in unbranded tins, and sent to the study locations. The snus tins were stored at 4°C until use and allowed to equilibrate to room temperature for approximately 15 min before use.

#### Study conditions

Each subject attended two sessions on different days. Subjects were recruited on the basis that they typically place their snus pouch under their upper lip, on either side of the mouth. At each session four snus pouches were used consecutively by each subject, with each pouch being kept in the mouth for 1 h. Subjects were asked to use the product in their normal manner, with no restriction on pouch movement. There was a 15 min break between pouches, during which time water, but no other beverage or food, could be consumed. A representative from the agency controlled the distribution and collection of samples. Subjects were asked not to use snus for at least 1 h before each session.

For each pouch used by a subject, a corresponding unused pouch was taken from the same tin. All used and unused pouches were immediately weighed and stored in individual sealed tubes of the same type. For the first session, pouches were stored in Class 200 pre-cleaned EPA glass vials (Greyhound Chromatography, Birkenhead, UK). For the second session, the first two used and unused pouches were stored in the glass vials, but the third and fourth used and unused pouches were stored in 50 mL plastic centrifuge tubes (Fisher Scientific, Loughborough, UK) to avoid contamination with sodium ions from the glass vials. All samples were stored at −20°C until analysis.

### Multi-analyte analysis of constituent extraction from pouched snus

#### Sample preparation

The snus samples were thawed at room temperature for 1 h prior to extraction. Unused pouches were extracted identically to the corresponding used pouch. To enable multiple analyses to be conducted on a single snus extract, validated in-house analytical methods were modified by changing the solvent volume, extraction method, or application of internal standard.

#### Methanol extraction and analysis

Methanol (HPLC grade, Rathburn Chemicals, Walkerburn, UK) was used to extract nicotine, tobacco-specific nitrosamines (TSNAs; n-nitrosoanabasine [NAB], n-nitrosoanatabine [NAT], n-nitrosonornicotine [NNN] and 4-(methylnitrosamino)-1-(3-pyridyl)-1-butanone [NNK]), propylene glycol and water. Whole pouches in their glass vials were extracted with 20 mL of dry methanol with shaking (180 rpm, 30 min). The solution was transferred to a 50 mL plastic centrifuge tube for centrifugation (4600 rpm, 5 min).

Nicotine levels were measured by gas chromatography (GC) using an HP6890 GC instrument fitted with an autosampler and 5973 mass selective detector (Agilent Technologies, Wokingham, UK). For analysis, an aliquot of the methanol-extracted supernatant was diluted 1:10 in methanol, and 10 μg/mL of d_4_-nicotine (99%, Toronto Research Chemicals, Ontario, Canada) was added as the internal standard. The resulting solution was injected (1 μL) into a splitless injector at 250°C. A 30 m × 0.25 mm ID × 0.25 μm GC column (J&W HP-5MS; Agilent Technologies) was used with a temperature gradient of 70°C to 230°C over 44 min and helium as the carrier gas at 1.5 mL/min. The mass selective detector was operated in SIM mode with a source temperature of 230°C and a quadrupole temperature of 150°C.

TSNA levels were measured by liquid chromatography tandem mass spectrometry (LC-MS/MS) using an API 5000 triple quadrupole MS/MS (Applied Biosystems, Warrington, UK) in positive ESI mode, with a mass/charge (*m*/*z*) range of 5–1250. For analysis, deuterated internal standards (5 ng/mL of d_4_-NAB; 10 ng/mL of d_4_-NAT, d_4_-NNN and d_4_-NNK; QMX Laboratories, Thaxted, UK) were added to an aliquot of the methanol-extracted supernatant. The resulting solution was injected (5 μl) onto a 1200 series LC system (Agilent Technologies), consisting of 1200 series binary pump, autosampler, vacuum degasser, column compartment and control module, with a Luna 3 μm C18(2) 100 Å, 100 mm × 2.00 mm column and a SecurityGuard cartridge kit (Phenomenex, Macclesfield, UK) as a guard column. The mobile phases were 5 mM aqueous ammonium acetate (99.99+%, Sigma-Aldrich, Gillingham, UK) and 5 mM ammonium acetate in 95% acetonitrile (HPLC grade, Rathburn Chemicals) and 5% water; the flow rate was 0.2 mL/min. The transition of the protonated parent/daughter-ion pairs *m/z* 192–162, *m/z* 190–79, *m/z* 208*–*122 and *m/z* 178–148 were monitored for NAB, NAT, NNK and NNN, respectively.

Propylene glycol was analyzed using an HP6890 GC fitted with a flame ionization detector (Agilent Technologies). For the analysis, 150 μg/mL of 1,3-butanediol internal standard (99+%, Sigma-Aldrich) was added to an aliquot of the methanol-extracted supernatant. The resulting solution (1 μL) was injected into a 980 μL split injector packed with quartz wool at a 20:1 split ratio. A DB5 30 m × 0.53 mm ID × 5.0 μm column (Agilent Technologies) was used with helium as the carrier gas at 16 mL/min. The temperature program was 60°C to 260°C over 14 min. The FID detector was operated at 275°C.

The water content of the methanol-extracted supernatant was determined using a Cary 5E double-beam near-infrared spectrometer (Varian Inc., Oxford, UK). The intensity of the combination band at 1943 nm (caused by −OH stretching and H−OH bending of the water molecule) was measured and compared with standard solutions of de-ionized water in methanol.

#### Water extraction and analysis

Water (de-ionized at 18.2 MΩ, Elga Process Water, High Wycombe, UK) was used to extract ammonium and nitrate nitrogen, and sodium and chloride ions. Whole pouches in their plastic tubes were extracted with 40 mL of water with shaking (180 rpm, 30 min) and then centrifuged (4600 rpm, 5 min). For sodium and chloride ion analysis, aliquots of the supernatant were diluted 1:10 in water.

Sodium ion content was measured by cation ion chromatography using an ICS-3000 ion chromatography system with an EGC II MSA eluent generator (Dionex UK Ltd, Camberley, UK) and a continuously regenerated cation trap column. A 25 μl aliquot was injected onto an Ionpac CG12A 2 mm × 50 mm guard column with an IonPac CS12A 2 mm × 250 mm analytical column. The mobile phase (flow rate 0.25 mL/min) was methane sulfonic acid. Detection was achieved via an Ultra II cation conductivity detector with a cation self-regenerating suppressor.

Chloride ion content was measured by anion ion chromatography using an ICS-3000 ion chromatography system with an EGC II KOH eluent generator (Dionex UK Ltd) and a continuously regenerated anion trap column. A 25 μl aliquot was injected onto an Ionpac AG15 2 mm × 50 mm guard column with an IonPac AS15 2 mm × 250 mm analytical column. Potassium hydroxide was used as the mobile phase (flow rate 0.30 mL/min). Detection was achieved via an Ultra II anion conductivity detector with an anion self-regenerating suppressor.

The nitrate content of the aqueous supernatant was measured by continuous flow analysis using a Series 2000 Analyzer (Burkard Scientific, Uxbridge, UK). Reduction of nitrate to nitrite with hydrazinium sulfate (AR grade, Fisher Scientific) was followed by reaction with sulfanilamide (GPR grade, Fisher Scientific) to form the diazo compound. This was then coupled with *N*-1-naphthyl-ethylenediamine dihydrochloride (GPR grade, Fisher Scientific) to form a colored complex, adsorption of which was measured at 550 nm in a flow cuvette.

The ammonia content of the aqueous supernatant was measured by continuous flow analysis using a Series 2000 Analyzer. The principle of the analysis is a modification of the Berthelot reaction, in which ammonia reacts with salicylate ions and hypochlorite to form indophenol blue and nitroprusside is added as catalyst. The indophenol blue color intensity was measured at 650 nm. All reagents were purchased from Fisher Scientific.

The pH of the aqueous supernatant was also measured, by using a Hanna 213 pH meter (Hanna Instruments Ltd, Leighton Buzzard, UK).

#### Ethanol extraction and analysis

Ethanol (AR grade, Fisher Scientific) was used to extract the analytes linalool, citronellol, linalyl acetate and geraniol. Whole pouches in their glass vials were extracted with 20 mL of ethanol with shaking (180 rpm, 30 min). The solution was transferred to a 50 mL plastic tube and centrifuged (4600 rpm, 5 min).

Linalool, citronellol, linalyl acetate and geraniol in the extract were analyzed by GC-MS using a Gerstel Multipurpose MPS-2 Twister Sampler (Anatune Ltd, Cambridge, UK) on a HP6890N GC with 5973 and 5975 mass spectral detectors (Agilent Technologies). Calibration was performed with matrix-matched standards in ethanol. Matrix-loaded ethanol was used to reduce liner activity in the GC. The matrix solution was prepared by adding 25 g of a snus tobacco blend (linalool-, citronellol-, linalyl acetate- and geraniol-free) to 500 mL of ethanol (Fisher Scientific), followed by shaking and decanting the ethanol from the tobacco and centrifuging. The supernatant from the ethanol-extracted pouches (1 μl) was injected in pulsed splitless mode (inlet temperature, 280°C; pressure, 7.84 psi; pulsed pressure, 20.0 psi for 2 min, followed by a purge flow of 25.0 mL/min for 2 min). A 30 m × 0.32 mm ID × 1.8 μm film RTX-VMS column (Thames Restek, Saunderton, UK) was used with helium as the carrier gas at 2.5 mL/min and a ramped temperature program from 60°C to 260°C over 36.1 min. The mass spectral detector was used in SIM mode.

### Validation of the analytical protocols

The modified multi-analyte approaches were first validated by comparing data obtained for LS Brown snus using the approaches described above with data previously obtained for this product using established in-house methods. The impact of saliva on the analysis of snus constituents was also evaluated. Saliva was obtained from a non-tobacco user and 0.3 mL was added to each of three unused snus pouches. The pouches were then extracted and analyzed by the methods described above and the results compared with data from the analysis of saliva-free unused pouches (N=5) of the same brand.

Lastly, the stability of the samples under the post-consumption storage conditions was assessed. Unused pouches (N=3) and unused pouches with added saliva (0.3 mL, N=3) were stored frozen (−20°C) for two weeks, and then allowed to thaw at room temperature for a minimum of 1 h prior to analysis. The data were compared with those for fresh unused pouches (N=5) and fresh unused pouches with added saliva (N=5). For TSNAs only, this was carried out with an increased number of pouches (N=15 in all cases).

### Calculation of constituent extraction by subjects

Values for the amount and percentage of a constituent extracted by the subject were calculated for each pair (used and unused) of samples according to the following equations:

$$\begin{array}{ll} \mathrm{Amount}\;\mathrm{extracted} & =\mathrm{Quantity}\;\mathrm{in}\;\mathrm{unused}\;\mathrm{pouch}\\ & -\mathrm{Quantity}\;\mathrm{in}\;\mathrm{used}\;\mathrm{pouch}\\ \mathrm{Percentage}\;\mathrm{extraction} & =100\times \left(\mathrm{Amount}\;\mathrm{extracted}\right)\\ & \left(\;/\mathrm{Quantity}\;\mathrm{in}\;\mathrm{unused}\;\mathrm{pouch}\right) \end{array}$$

### Statistical analysis

All analytical data were generated on samples as received with no correction for pouch mass or water content (i.e. wet-weight basis). The methanol- and ethanol-extracted analytes were analyzed in triplicate (one measurement per pouch, three pouches per subject). The water-extracted analytes were measured in duplicate (one measurement per pouch, two pouches per subject).

Minitab version 15 (Coventry, UK) was used for statistical analysis. Two-sample *t*-tests were used to compare the amount of analyte between used and unused pouches. A value of *p*<0.05 was taken to be significant. The distribution of percentage extraction values was approximately normal for all analytes except geraniol and citronellol; however, transformation of the latter data had no significant effect on analysis. As a result, all analyses were performed on non-transformed data.

For each constituent, total variability was calculated as the coefficient of variation in percentage extraction. To determine measurement variability (a combination of product and analytical variation), the standard deviation (SD) of analyte content in unused pouches was used. Because two pouches (the used and unused pair) were analyzed to calculate extraction values, the pooled SD (square root of twice the square of the SD) was used to calculate the variation coefficient, with the resulting percentage indicating the contribution of measurement variability to total variability. Fully-nested analysis of variance (ANOVA) was used to estimate the contribution of intra- and inter-subject variability to total variability.

## Results

### Validation of the study design and analytical approach

Unpublished data from our previous telephone survey [7] showed that 79.5% of pouched snus users stated that they had never swallowed a pouch and always removed it after use, and 19.3% stated that swallowing had occurred unintentionally at some time. Only a minority (1.2%) had ever intentionally swallowed a pouch. These data supported the study approach in which subjects used each pouched snus product for 1 h before removing it for analysis.

The multi-analyte approach was validated by comparing the data obtained from each modified method with data obtained from the corresponding established method. The two sets of data were in good agreement (Table 1). A significant difference was seen between the two data sets for nicotine, nitrate and pH (*p*<0.05). For both nitrate and pH, however, the percentage difference between the two methods (2.6% and 0.5%, respectively) was deemed insignificant in practical terms. The percentage difference between the two methods for nicotine (20.2%) resulted from a difference in analytical procedure: in the established method, nicotine was determined in tobacco removed from the snus pouch; in the multi-analyte method, nicotine was determined in the whole pouch. Analysis of the pouch material without tobacco revealed that approximately 1 mg of nicotine was present in the pouch material itself, which corresponds to the difference between the established and multi-analyte methods. Although 20% presence of nicotine in the pouch material is surprisingly high, repeated analysis (data not shown) confirmed the presence of nicotine in the pouch material. The measured nicotine in the pouch material in this repeated analysis was equivalent to 0.95 mg or 11% of the total nicotine in the snus portion.

**Table 1:** Comparison of multi-analyte and established analysis methods

**Analyte**	**Mean ± SD value from established method, per gram basis *(N=5)***	**Mean ± SD value from multi-analyte method, per whole 1 g pouch basis *(N=5)***	**Percentage change relative to established method**	***p *value**
**Water (%)**	48.7±1.38	49.4±0.90	1.4	0.391
**NAB (ng)**	22.7±0.66	21.6±1.05	−4.8	0.092
**NAT (ng)**	377.7±5.26	382.2±5.91	1.2	0.246
**NNK (ng)**	166.1±4.25	165.2±6.19	−0.5	0.783
**NNN (ng)**	498.9±18.03	495.6±23.10	−0.7	0.811
**Nicotine (mg)**	8.2±1.00	9.9±0.13	20.2	0.021
**Propylene glycol (mg)**	35.7±0.58	35.3±0.43	−1.1	0.258
**Sodium (mg)**	21.5±0.29	21.3±0.75	−0.9	0.610
**Chloride (mg)**	29.8±1.18	29.1±1.57	−2.3	0.486
**Ammonium (μg)**	1358±32.0	1406±39.3	3.5	0.071
**Nitrate (μg)**	1500±16.6	1462±12.8	−2.6	0.004
**pH**	8.5±0.02	8.5±0.01	0.5	0.002

Regarding the effect of saliva, a higher water content (81.6% compared to 49.0%) and slightly lower pH value (8.3 compared to 8.5) were found for samples with added saliva. Significant decreases (*p*<0.05) ranging from 6.5% to 12.5% in sodium, chloride, ammonium and nitrate were observed for the pouches with added saliva. The amounts of these analytes were also lower than those in the fresh pouches with added saliva analyzed in the frozen storage trial, which themselves were not significantly different (*p*>0.05) from fresh, unaltered pouches. Considering this, and the fact that aliquots for each of these four analytes were taken from the same water extract, the differences observed were considered to have arisen from measurement (potentially extraction volume) error.

With regard to post-consumption storage conditions, significant differences (*p*<0.05) between the results for fresh and those for frozen pouches with added saliva were seen for pH and geraniol, but these small reductions (1.3% and 3.4%, respectively) were considered to be not significant in practical terms. Significant differences were also seen between frozen unaltered pouches and fresh unaltered pouches for propylene glycol, linalool and linalyl acetate. Again, the changes were small (5.6%, 4.4% and 5.6%, respectively) and considered to be insignificant in practical terms.

No significant difference was found in the NAB, NAT and NNN content of pouches stored frozen for 2 weeks relative to fresh pouches, for both saliva-added and unaltered pouches, nor in the NNK content of unaltered pouches (*p*>0.05 in each case). A statistically-significant difference found in the NNK content of pouches stored frozen for 2 weeks with added saliva compared to fresh pouches with added saliva (*p*=0.048, 4.3%) was considered to arise from measurement (product and analytical) variation.

### Snus properties before and after use

The mean unused sample weight for pouched snus samples was 1.0 ± 0.04 g and these were selected freely from the tins of product during the study. During use, the weight of snus pouches increased by a mean of 0.26 ± 0.08 g, mainly through a 29.0% increase in water content. This corresponds to a 58.4% increase relative to the mean unused pouch water content of 49.7%. Mean sample pH fell marginally from 8.2 to 8.0, most probably because of increased saliva levels and salivary pH, which ranges from approximately 6.5 – 7.5 [15], being lower than that of the snus.

### Extraction of tobacco constituents by subjects

The amount and percentage extraction of constituents by subjects during use are given in Table 2. While the constituent level in the snus pouches varied from nanogram (e.g. TSNAs) to milligram (e.g. nicotine, sodium and propylene glycol) quantities, the mean percentage extracted varied from 19.2% (linalyl acetate) to 37.8% (NNK) among all constituents analyzed. Notably, the TSNAs, some of which are known carcinogens [1], showed the highest percentage extraction (mean [median] values: NAT, 34.6% [34.6%]; NNK, 37.8% [37.8%]; NNN, 35.6% [35.3%]; NAB, 36.3% [36.7%]; total TSNAs, 35.8% [35.9%]).

**Table 2:** Extraction of constituents from pouched snus by subjects

**Constituent**	**Mean ± SD amount in unused product**	**Mean ± SD amount in used product**	**Mean ± SD amount extracted**	**Mean ± SD percentage extracted**	**Median percentage extracted**	**Number of pouches analyzed**
**Chloride (mg/pouch)**	35.3±2.23	25.5±4.59	9.8±4.10	27.7±11.28	28.7	41
**Propylene glycol (mg/pouch)**	31.1±1.97	21.9±3.75	9.2±3.35	29.7±10.62	30.3	63
**Sodium (mg/pouch)**	24.7±1.97	18.6±3.15	6.1±2.91	24.8±11.21	25.9	41
**Nicotine (mg/pouch)**	9.6±0.90	6.4±1.12	3.2±1.00	33.3±9.86	32.7	63
**Ammonium (μg/pouch)**	1283.7±98.10	919.0±142.45	364.7±136.59	28.3±9.88	29.8	42
**Nitrate (μg/pouch)**	1215.3±88.13	892.3±145.52	323.0±132.97	26.6±10.50	26.2	42
**Linalyl acetate (μg/pouch)**	150.1±12.3	121.0±21.38	29.1±20.58	19.2±13.49	18.9	63
**Geraniol (μg/pouch)**	15.4±1.70	11.6±1.42	3.81±2.22	23.7±14.40	27.6	63
**Linalool (μg/pouch)**	148.8±12.87	103.2±14.40	45.6±15.77	30.4±9.65	29.8	63
**Citronellol (μg/pouch)**	32.5±3.06	23.3±4.48	9.2±4.64	28.2±13.24	28.4	63
**Total TSNAs (ng/pouch)**	830.0±64.16	532.1±74.40	297.9±74.88	35.8±7.85	35.9	63
**NAT (ng/pouch)**	268.7±20.50	175.6±23.62	93.0±23.30	34.6±7.65	34.6	63
**NNK (ng/pouch)**	191.8±19.66	118.9±18.41	72.8±19.47	37.8±8.10	37.8	63
**NNN (ng/pouch)**	344.4±29.79	221.6±33.99	122.8±32.97	35.6±8.46	35.3	63
**NAB (ng/pouch)**	25.1±2.39	15.9±2.02	9.2±2.47	36.3±7.72	36.7	63

### Variability of results

Table 3 shows the total variability in percentage extraction for each constituent and the contribution of measurement, intra- and inter-subject variability to this total.

**Table 3:** Variability in percentage extraction of constituents from pouched snus

**Constituent**	**Mean ± SD extraction (%)**	**Total variability (%)**	**Measurement variability (%)**	**Intra-subject variability (%)**	**Inter-subject variability (%)**
**Chloride**	27.7±11.28	40.7	3.6	12.8	24.3
**Propylene glycol**	29.7±10.62	35.8	3.2	8.8	23.8
**Sodium**	24.8±11.21	45.2	5.1	15.2	24.9
**Nicotine**	33.3±9.86	29.6	3.9	16.2	9.5
**Ammonium**	28.3±9.88	34.9	3.8	14.0	17.1
**Nitrate**	26.6±10.50	39.5	4.1	11.5	23.9
**Linalyl acetate**	19.2±13.49	70.3	8.1	10.0	52.2
**Geraniol**	23.7±14.40	60.8	9.5	29.7	21.6
**Linalool**	30.4±9.65	31.7	3.9	3.7	24.1
**Citronellol**	28.2±13.24	47.0	6.3	3.4	37.3
**Total TSNAs**	35.8±7.85	21.9	2.4	10.5	9.0
**NAT**	34.6±7.65	22.1	2.4	11.2	8.5
**NNK**	37.8±8.10	21.4	3.1	10.9	7.4
**NNN**	35.6±8.46	23.8	2.9	10.6	10.3
**NAB**	36.3±7.72	21.3	2.9	11.7	6.7

The total variability ranged from 21.3% (NAB) to 70.3% (linalyl acetate). Measurement variability was low for all analytes, ranging from 2.4% (total TSNAs, NAT) to 9.5% (geraniol). There was a greater spread of intra-subject variability, ranging from 3.5% (citronellol) to 29.7% (geraniol). The intra-subject variability for geraniol was markedly higher than that for any other analyte, the next highest value being 16.2% for nicotine. Inter-subject variability ranged from 6.7% (NAB) to 52.2% (linalyl acetate); this type of variability was generally much higher, being higher than 20% for 8 of the constituents analyzed.

## Discussion

This study has examined the extent of and variability in constituent extraction from Swedish pouched snus during normal consumption. The technique used in this study for estimating tobacco constituent exposure relies on subjects removing the pouch after use and not expectorating salivary leachates during use. Snus use in Sweden is “spitless” and users do not expectorate during use; furthermore, only 1.2% of survey respondents stated that they had ever intentionally swallowed a snus pouch. As a result, this approach was considered valid in the context of snus use in Sweden.

The multi-analyte extraction methods developed for this study are flexible in that they allow examination of the extraction characteristics of one or more of several constituents from the same snus pouch. This maximizes the amount of data that can be obtained from a minimum number of samples and subjects. Validated against established in-house methods, the changes made to harmonize analytical protocols did not significantly affect measurement of any of the analytes. Whole-pouch extraction in the collection vial removed the need for further handling of used snus pouches; furthermore, inclusion of the pouch material during extraction ensured that any analytes absorbed therein, such as nicotine, were included in the analysis.

The levels of extraction of constituents from pouched snus were generally low. For most constituents, less than a third was extracted during use. The present findings are in good agreement with five previous studies of snus extraction. Andersson et al. examined the extraction of tobacco constituents by 23 users of pouched snus [12]. The subjects used snus in their normal manner and collected all samples used in 1 day. Because the samples were combined, the study design did not permit examination of pouch-to-pouch variation during the day. On a percentage basis, subjects extracted 37.4 ± 17.6% of the nicotine and 55.7 ± 20.5% of the TSNAs. Although the level of nicotine extraction was similar, TSNA extraction was lower in the present study. However, the uncontrolled conditions of snus use in Andersson et al.’s study contrasts with the controlled 1 h use in our study, which may account for the differences in results between the two studies.

Lunell and Lunell studied the extraction of nicotine and sodium chloride from four brands of pouched snus by 12 male users who placed the pouch in their mouth for 30 min [10]. The subjects extracted 31% (2.74 ± 0.18 mg) of the total nicotine from 1 g pouches of a “brown”-style product, which is consistent with the present study result of 33 ± 9.9% for LS Brown, despite the difference in usage duration (30 min versus 1 h). Nicotine extraction from 1 g pouches of a “white”-style product (1.55 ± 0.18 mg; 22% of total) was lower, and that from 0.5 g pouches of another “brown”-style product was more efficient (2.00 ± 0.11 mg; 44% of total). Between 4.73 ± 6.61 and 10.38 ± 6.83 mg of salt per pouch was extracted. The greater extraction of sodium chloride in the present study (15.5 mg) may reflect the longer duration of usage.

In a follow-up study, Lunell and Lunell reported the extraction of nicotine, TSNAs and metals from the same four brands by 32 male snus users [16]. Up to 30% of nicotine was extracted from the pouches, and TSNA extraction was similar. The slightly greater mean extraction of nicotine (33.3%) and TSNAs (35.8%) found in the present study may be an effect of the increased usage duration (1 h compared to 30 min), but are generally in agreement with these observations.

Lunell and Curvall later examined nicotine extraction from two pouched snus products used for 30 min [13]. The smaller mean extraction observed (21%–25%), as compared with their previous study (22%–44%) [10], was suggested to be due to the recruitment of smokers rather than experienced snus users. In our study, experienced snus users were recruited, similar to the initial study of Lunell and Lunell [10]. Thus, the higher mean nicotine extraction of 33% that we observed might reflect the greater usage time (1 h) and differences among study subjects. The effect of usage time on the extent of extraction is an important area for future examination.

Most recently, Caraway and Chen studied constituent extraction by 53 users of three similar U.S. pouched snus products [14]. Despite almost identical baseline levels of total TSNAs, a lower mean extraction was observed (21.6 ± 21.4%) compared to our study (35.8 ± 7.9%). This may again reflect the reduced usage time (three-quarters of the subjects stated that they used each pouch for 30 min or less), but may also have arisen due to product differences such as water content (32%, compared to LS Brown at 52%). Also storage of used pouches at ambient temperatures during the day of collection may have led to TSNA formation and thus lower extraction values. Mean extraction of nicotine (39.2 ± 23.0%) was slightly higher than in our study (33.3 ± 9.9%) and substantially more variable. This higher degree of variability, which is also reflected in the TSNA values, is likely to have arisen due to the ambulatory nature of the study, compared to the controlled conditions which we employed.

Our results, as well as others [10,12,14], show that extraction of snus constituents by users is a variable process, with SDs ranging from approximately 20% to 70% of the mean percentage extraction (Table 3). The inability to compare the constituent content of a single pouch before and after use introduces errors due to the variability of unused pouches. For example, if the nicotine content of two similar unused pouches differed by 1SD (0.9 mg; Table 2) and one was assumed to have been used, this would result in a calculated percentage extraction of 9.4% even in the absence of any use by subjects. To our knowledge, there is no non-destructive method to analyze the chemical content of a snus pouch before use, and thus to eliminate this source of variation, without rendering it unusable by study subjects.

This degree of variability is substantial and has been shown to encompass the measurement (i.e. unused product and analytical method), and both intra- and inter-subject variability (Table 3). For almost all analytes, measurement variability was the smallest component of the total variation. For nicotine, TSNAs and geraniol, intra-subject variability was slightly greater than inter-subject variability. For the remaining constituents, inter-subject variability made the greatest contribution to total variation.

Measurement variability is a composite of product variability, arising from tobacco blend and ingredient heterogeneity, and analytical variation resulting from operator, reagent and machinery sources. Intra-subject variability is likely to arise from factors influencing a subject’s extraction of constituents from snus, such as saliva composition and rate of secretion, pressure applied to the pouch with the lip and/or gum during use, and movement of the pouch during use. Inter-subject variability may be affected by the same factors, as well as by the different physiological characteristics of subjects.

**Figure 1:**
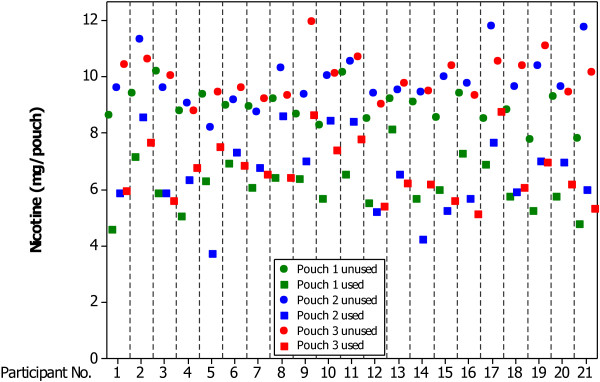
**Individual values of the nicotine content in unused and used pouches according to subject number.** For each subject, three single measurements were made from each of three used and three unused pouches.

Figure 1 shows the variability of nicotine content of both unused and used pouches, on an inter- and intra-subject basis. For each subject, the residual nicotine content of each of the three used pouches was lower than the nicotine content of the three corresponding unused pouches, demonstrating that nicotine was extracted by each subject. ANOVA analysis confirmed no significant difference in the extent of extraction from pouches 1, 2 and 3 (*p*>0.05*)*. Some subjects (e.g. numbers 3, 6, 7) were relatively consistent in the amount of nicotine that they extracted, whereas others (e.g. numbers 5, 13, 16) were relatively inconsistent. Measurement variability is exemplified by the intra-subject spread in unused pouch nicotine content, but inter- and intra- subject variability in the residual nicotine content of used pouches is clearly greater. Analysis of these data indicates that intra-subject variability is greater than inter-subject variability, whereas measurement variability contributes the least to overall variation.

Given these levels of variation, it is valuable to establish the number of subjects needed to observe a significant difference for future studies. Two-sample *t*-test power calculations (α=0.8) were performed using the present data. The numbers of subjects (95% CI) needed to observe a 1%, 10% or 30% difference in nicotine extraction between two snus pouches would be 1528, 17 or 4, respectively. Similarly, to observe a difference in TSNA extraction of 1%, 10% or 30% would require subject numbers of 969, 11 or 3, respectively.

**Figure 2:**
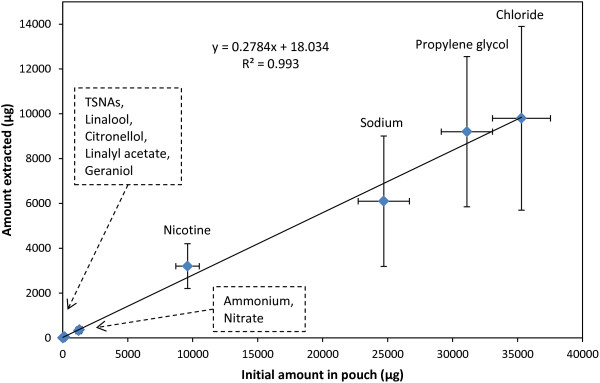
**Extraction of constituents from pouched snus with increasing initial amount present in pouch.** For each analyte, the mean value of the amount extracted is plotted against the mean value of the amount in unused pouches (Table 2). Error bars represent the SD.

We also investigated the importance of constituent concentration on extraction efficiency. The absolute amount of a given constituent extracted from a pouch during typical use seems to be dependent on the amount of that material in the pouch before use (Figure 2). The percentage extraction of water-miscible compounds was in the same range whether the constituent was present in milligram (e.g. nicotine) or nanogram (e.g. NNN, NNK) quantities. This agreement, despite the 1-million-fold difference in levels, suggests that there is a common mechanism of constituent extraction in which the chemistry of the constituent does not have a significant role. These observations are consistent with the rate-limiting step for extraction being the supply of saliva to the snus pouch in the user’s mouth. The fast, rich blood flow in the buccal mucosa (region where the snus pouch is placed) has been reported to facilitate passive diffusion of drug molecules across this membrane [17]. However; it is likely that dissolution of snus constituents into the thin layer of saliva coating this membrane must occur first. Clearly, this hypothesis needs to be tested in future trials with appropriate samples.

**Figure 3:**
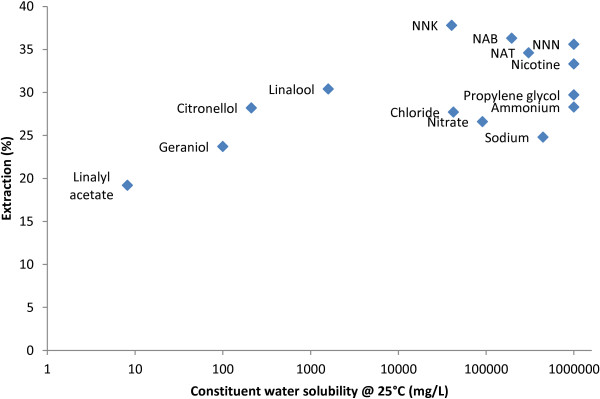
**Extraction of constituents from pouched snus by subjects versus constituent water solubility.** Note that the horizontal scale is logarithmic. Values for the water solubility of constituents were obtained from the SRC Inc. Online Interactive Demo Version of the Physical Properties Database [20] and the Royal Society of Chemistry ChemSpider database [21].

Constituents may be transferred across the buccal mucosa by various processes, including passive or facilitated diffusion from an area of high (snus pouch) to low (buccal mucosa) concentration, active transport and endocytosis [18]. Physicochemical factors such as molecular size and hydrophilic/lipophilic nature of a given constituent determine whether transport occurs via one or a combination of these processes, and therefore the net rate with which transport occurs [17]. Notably, the constituent showing the lowest percentage extraction, linalyl acetate (19.2%, Table 2), is also the least water-soluble of those investigated. It has been reported that the extraction of cadmium from snus was less than 10%, and that lead extraction was negligible [16]. A recent study has shown that a significant proportion of the arsenic in tobacco is insoluble in water, and that the arsenic that is soluble comprises a range of species [19]. Hence, the low extraction of metals by subjects [16] may be a reflection of their low solubility in saliva. In our study, geraniol and citronellol, which are also very sparingly soluble in water, were extracted to a greater extent (23.7% and 28.2%, respectively) than linalyl acetate. The present data suggest that the saliva and/or water solubility of a constituent influences its extraction (Figure 3); as a result, it might be expected that the other constituents examined here, which are strongly water soluble, would be extracted to a greater extent. This was the case for nicotine, propylene glycol and the TSNAs, which were extracted to a similar extent; however, the percentage extraction of sodium, chloride, ammonium and nitrate was slightly lower. However, it must be noted that most analytes were extracted with reasonably similar efficiency, despite substantially different water solubility. The effect of saliva solubility is relatively weak in the present study and requires more extensive studies to examine its influence.

The present methodology provides an effective approach for estimating tobacco constituent extraction by snus users. In theory, this methodology could be applied to any constituent for which an analytical method has been established, as long as neither human saliva nor the storage/transport conditions affect the stability of that constituent. In addition, this approach might be extended to other types of STP for which there is sufficient knowledge of normal/average usage behavior and patterns. Important considerations would include average portion size, typical duration of use, location of the STP in the mouth, mechanical work (e.g. chewing) performed by the user during use, and residue (saliva and tobacco) removal habits after use.

The results of this study, and others [10,12-14,16], indicate that further research is needed in this area. For example, to understand more fully both the importance of usage time and the possible consequences of extreme usage durations on snus constituent extraction would require a dedicated time-course study covering usage durations from 20 to 120 min [7]. The influence of constituent quantity and saliva solubility on extraction might also be tested, as well as the effects of product parameters such as pouch size and water content. To further inform understanding of constituent exposure, the impact of real-life factors should also be considered. Under conditions of everyday use, for example, snus users may typically consume beverages or food while using snus, and such practices may affect constituent extraction.

In summary, we have described an approach for investigating constituent extraction from pouched snus. A pilot study conducted using this approach has provided insight into the extent of constituent extraction during human use. The values reported for nicotine, TSNAs and sodium chloride confirm that, for the majority of constituents tested, only a small proportion is extracted during use. The data indicate the importance of constituent quantity, and the possible role of constituent solubility and duration of usage, on the amount extracted. Lastly, this study has given insight into possible mechanisms controlling snus extraction in the mouth and provides valuable reference data for the development of in-vitro laboratory systems for estimating exposure to tobacco constituents in snus [22].

## Conclusion

In summary, a multi-analyte approach to the measurement of various tobacco constituents in a pouched snus sample has been developed and validated. This multi-analyte approach showed that generally less than a third of each constituent tested was extracted by a consumer during one hour of snus use, independent of the absolute constituent concentration. The variable nature of constituent extraction by snus users was found to be driven by inter-user variability. This study provides insight into possible mechanisms controlling constituent extraction in the mouth during snus use, and provides reference data for the development of in-vitro laboratory systems for estimating the extraction of tobacco constituents from snus.

## Competing interests

All authors are employees of British American Tobacco, which is a producer of snus.

## Authors’ contributions

HD participated in the design of and coordinated the study. NG performed data collation and analysis and drafted the manuscript. GE participated in the experimental design of the study and performed statistical analysis. NP developed the ethanol extraction and analysis method and performed these analyses. KMcA provided expert scientific advice, coordinated the validation of the multi-analyte analysis methods and drafted the manuscript. All authors read and approved the final manuscript.
